# The Alternative Pathway of Complement Activation May Be Involved in the Renal Damage of Human Anti-Glomerular Basement Membrane Disease

**DOI:** 10.1371/journal.pone.0091250

**Published:** 2014-03-21

**Authors:** Rui Ma, Zhao Cui, Shui-Yi Hu, Xiao-Yu Jia, Rui Yang, Xin Zheng, Jie Ao, Gang Liu, Yun-Hua Liao, Ming-Hui Zhao

**Affiliations:** 1 Renal Division, Department of Medicine, Peking University First Hospital, Peking University Institute of Nephrology, Key Laboratory of Renal Disease, Ministry of Health of China, Key Laboratory of Chronic Kidney Disease Prevention and Treatment (Peking University), Ministry of Education of China, Beijing, China; 2 Renal Division, Department of Medicine, First Affiliated Hospital of Guangxi Medical University, Nanning, Guangxi Zhuang Autonomous Region, China; 3 Peking-Tsinghua Center for Life Sciences, Beijing, China; University Medical Center Utrecht, Netherlands

## Abstract

Linear deposition of IgG and complement 3 (C3) along glomerular basement membrane (GBM) is generally revealed in the kidneys of human anti-GBM disease. Our recent studies demonstrated the pathogenic role of complement activation in renal damage of this disease. However, the pathways of complement activation were still paradoxical. In this study, renal biopsy tissues from 10 patients with anti-GBM disease were used to investigate the pathways of complement activation by detecting the deposition of various complement components, including C1q, factor B, factor P (properdin), mannose-binding lectin (MBL), C3d, C4d and C5b-9, using immunohistochemistry and immunofluorescence. We found that C1q, factor B, properdin, C3d, C4d and C5b-9 were detected in all the glomeruli of our patients, along GBM with a linear and/or granular staining pattern. Furthermore, C1q, factor B and properdin co-localized well with C5b-9. The properdin also co-localized well with C3d. However, the deposition of MBL was diffusive in mesangium, GBM, Bowman's capsule and within crescents and was not co-localized with C5b-9 but partially co-localized with C4d. The intensity of factor B deposition (3.3 vs. 1.2, *P*<0.001) and C5b-9 deposition (3.2 vs. 1.6, *P*<0.001) was significantly stronger in the glomeruli with crescent formation, compared with the glomeruli without crescents. The complement system is overall activated via both the alternative pathway and classical pathway in the kidneys of human anti-GBM disease. The alternative pathway might play an important role in complement activation induced renal damage.

## Introduction

Anti-glomerular basement membrane (GBM) disease is a rare but life-threatening autoimmune disease, which clinically manifests rapidly progressive glomerulonephritis with or without pulmonary hemorrhage [1], [2]. IgG autoantibodies against the non-collagenous domain of α3 chain of type IV collagen on GBM [α3(IV)NC1] have been proven to be pathogenic in the disease [3], [4]. The presence of circulating anti-GBM autoantibodies and linear deposition of IgG along glomerular capillary wall (GCW) are the characteristics of this disease [2]. In the renal biopsy of patients, linear deposition of IgG is often accompanied by complement 3 (C3) deposits, also on GCW in linear or granular staining pattern, which indicates that complement activation is involved in the kidney injury [5]. Our recent study in human anti-GBM disease also identified that the plasma level of the terminal component of complement activation, C5b-9, also named as membrane attack complex, was closely associated with the severity of kidney damage and was the predictor for renal failure during patient follow up [6].

The pathways of complement activation in anti-GBM disease are largely studied by passive injection of heterologous antibodies against GBM. This model in mice with complete deficiency of C3 or C4 revealed a protective effect of C3 deficiency more than that of C4 deficiency, which suggests the involvement of classical pathway and/or lectin pathway, and the involvement of alternative pathway in the complement activation [7], [8], [9]. However, none of the three pathways is clearly identified or excluded. In human anti-GBM disease, the pathways of complement activation are also poorly understood. Although anti-GBM disease is a well-known autoantibody induced disease, C1q, the key component of classical pathway, is seldom found deposit in the renal biopsy specimens [5]. In the present study, we investigated the deposition of various complement components in the renal biopsy specimens from patients with anti-GBM disease, with the aim to clarify the pathways of complement activation in the kidney injury of human anti-GBM disease.

## Materials and Methods

### Patients

Consecutive renal biopsy tissues were collected from 10 patients with anti-GBM disease admitted in Peking University First Hospital from 2002 to 2007. All the 10 patients had a positive test for circulating anti-GBM autoantibodies by enzyme-linked immunosorbent assay (ELISA) using purified bovine α(IV)NC1 as solid phase antigen, with confirmation of antibody specificity by ELISA against recombinant human α3(IV)NC1. Patients with any other coexisting diseases such as membranous nephropathy were excluded. The clinical data were collected from medical records. As complement activation has been considered to be not involved in minimal change disease, renal biopsy specimens from 5 such patients were used as disease control. Renal tissues obtained from the normal part of a nephrectomized kidney due to renal carcinoma were used as normal control. Each patient gave written inform consent when renal biopsy was performed and this study was in compliance of the Declaration of Helsinki and approved by the ethics committee of Pecking University first hospital.

### Renal Histopathology

Renal biopsy was performed at the time of diagnosis. For light microscopy, paraffin sections were stained with haematoxylin and eosin, periodic acid-schiff, periodic acid-silver methenamine and Masson's trichrome. For electron microscopy, biopsy materials were fixed in glutaraldehyde, postfixed in osmium tetroxide, dehydrated in graded acetone and embedded in Epon 812 resin. Ultrathin sections were stained with uranyl acetate and lead citrate, and examined by a transmission electron microscope JEM-1230 (JEOL, Tokyo, Japan). For direct immunofluorescence, frozen sections were examined by fluorescent microscope (Nikon, Tokyo, Japan) after staining with fluorescein isothiocyanate (FITC) -conjugated antibodies specific for human IgG, IgM, IgA, C3c, C1q, fibrinogen and albumin (Dako A/S, Copenhagen, Denmark).

### Detection of Renal Deposition of Complement Components by Immunohistochemistry

Staining of single complement component was performed by immunohistochemistry as our previously described [10]. To investigate the complement activation, immunohistochemical staining was performed for C1q, factor B, C3d, C4d and C5b-9 using primary antibodies listed in Table 1. 10% formalin-fixed, paraffin embedded renal tissues was cut into 4 µm sections. After deparaffinized in xylene and rehydrated in grading alcohols, sections were incubated in 3% H_2_O_2_ for 10 min at room temperature and in dark to quench endogenous peroxidase activity. Appropriate pretreatments for antigen retrieval were predetermined. Pepsin pretreatment consisted of 0.4% pepsin (Zhongshan Golden Bridge Biotechnology, Beijing, China) to sections for 45 min at 37°C. Proteinase K pretreatment consisted of 0.5 mg/ml proteinase K (Roche, Switzerland) to sections for 10 min at 37°C. After pretreatments, all sections were blocked non-specific staining for 30 min at room temperature with 3% bovine serum albumin, and then incubated with primary antibody overnight at 4°C. All the antibodies' optimal dilutions were predetermined by means of titration on renal sections from patients with lupus nephritis and IgA nephropathy. Dako EnVision HRP (Dako A/S, Copenhagen, Denmark) was used as detection system, which was an avidin-free two-step indirect method with goat anti-rabbit and goat anti-mouse immunoglobulins conjugated with horseradish peroxidase as secondary antibodies. The secondary antibodies were incubated for 30 min at 37°C and the sections were developed in fresh hydrogen peroxide plus 3-3-diaminobenzidine tetrahydrochloride solution for 1 min. Finally, the sections were counterstained with hematoxylin, differentiated with acid alcohol, dehydrated and cleared in alcohols and xylene, and coverslipped with permount. The sections were examined by light microscopy. As negative controls, normal rabbit IgG or normal mouse IgG replaced the primary antibodies.

**Table 1:** The species, dilutions and sources of primary antibodies for the detection of each complement component.

Complements	Species	Dilutions	Sources
C1q	Mouse anti-human monoclonal	1∶4000 (IHC)	Abcam, Cambridge, UK
		1∶400 (IF)	
Factor B	Mouse anti-human monoclonal	1∶50	Quidel, San Diego, CA, USA
Properdin	Mouse anti-human monoclonal	1∶50	Lifespan Biosciences, WA, USA
MBL	Mouse anti-human monoclonal	1∶30	Abcam, Cambridge, UK
C3d	Rabbit anti-human polyclonal	1∶1000 (IHC)	Dako A/S, Copenhagen, Denmark
		1∶100 (IF)	
C4d	Rabbit anti-human polyclonal	1∶400 (IHC)	Abcam, Cambridge, UK
		1∶40 (IF)	
C5b-9	Mouse anti-human monoclonal	1∶50 (IHC)	Abcam, Cambridge, UK
	Rabbit anti-human polyclonal	1∶20 (IF)	

MBL: mannose-binding lectin, IHC: immunohistochemistry, IF: immunofluorescence.

### Detection of Renal Deposition of Complement Components by Immunofluorescence Using Laser Scanning Confocal Microscopy

Sections were cut into 5 µm slides from frozen renal biopsy tissues and then air-dried for 25 min at room temperature and fixed in −20°C pre-cooled acetone for 10 min at 4°C. After washed three times with phosphate buffered saline, sections were incubated in 3% bovine serum albumin for 30 min at room temperature. Mouse antibodies against C1q, factor B, properdin and MBL were incubated overnight at 4°C as primary antibodies, respectively (Table 1). FITC-labeled goat anti-mouse IgG (Zhongshan Golden Bridge Biotechnology, Beijing, China) diluted 1∶30, was used as secondary antibodies for 30 min at 37°C. Next, rabbit anti-C5b-9 antibodies were added to the sections with C1q, factor B, properdin and MBL; rabbit anti-C3d antibodies were added to the sections with properdin; rabbit anti-C4d antibodies were added to the sections with MBL for 60 min at 37°C (Table 1). Tetramethylrhodamine isothiocyanate (TRITC) -labeled goat anti-rabbit IgG (Zhongshan Golden Bridge Biotechnology, Beijing, China) diluted 1∶30, was used as secondary antibodies for 30 min at 37°C. The sections were air-dried in dark, mounted with citifluor (Zhongshan Golden Bridge Biotechnology, Beijing, China), and visualised using a fluorescence microscopy (Nikon 80i, Tokyo, Japan) and a confocal microscope (Olympus viewer 1000, Tokyo, Japan). Renal tissue sections from patients with lupus nephritis and IgA nephropathy were used as positive controls. As negative controls, normal rabbit IgG or normal mouse IgG replaced the primary antibodies.

### Criteria for Semiquantitative Scoring

Two pathologists evaluated all sections separately, blinded to each other and the patients' data. Differences in scoring between the two pathologists were resolved by re-reviewing the sections and coming to a consensus.

The intensity of GCW staining of complement components was evaluated at 400× magnification and scored on a scale of 0 to 4+: 0, no staining; 1, mild staining; 2, moderate staining; 3, moderate-high staining; and 4, high staining on a high-power field.

Co-localization of different complement components was judged by merging of the green fluorescence of FITC and the red fluorescence of TRITC.

### Statistical Analysis

Differences in quantitative parameters between groups were assessed using Student *t*-test (for normally distributed data) or nonparametric test (for non-normally distributed data). Differences in semiquantitative data were tested using Kruskal Wallis H one-way analysis and Mann-Whitney U test. Differences in qualitative data were compared using Chi square test. Pearson's correlation test was used to measure the correlation between two normally distributed variables. Spearman's correlation test was used to measure the correlation between two non-normally distributed variables or one normally with one non-normally distributed variable. All statistical analyses were two-tailed and *P*<0.05 was considered as significant. Analysis was performed using SPSS statistical software package, version 13.0 (SPSS Inc., Chicago, IL, USA).

## Results

### Demographic, clinical and pathological data of patients with anti-GBM disease

The demographic, clinical and pathological parameters of the 10 patients with anti-GBM disease were listed in Table 2. Among them, 7 were male and 3 were female with a median age of 23 years (range, 20–66 years). 6 (60%) patients had pulmonary hemorrhage and presented as Goodpasture syndrome. The mean level of serum creatinine was 557.4±224.2 µmol/L at presentation. 2 (20%) patients had positive ANCA, both with specificity for MPO.

**Table 2:** The demographic, clinical and pathological parameters of patients with anti-GBM disease.

Patient	Gender	Age	pulmonary hemorrhage	SCr (µmol/L)	Anti-GBM antibodies in circulation (U/mL)	ANCA	Percentage of crescents in glomeruli	Routine direct immunofluorescence						
								IgG	IgA	IgM	C3c	C1q	FRA	Alb
1	Male	23	+	581	33	−	100%	3+ (GCW)	−	−	−	−	−	−
2	Male	23	+	224	13	−	90%	3+ (GCW)	−	−	3+ (GCW)	−	−	−
3	Male	20	+	551	21	−	100%	3+ (GCW)	−	−	3+ (GCW)	−	−	−
4	Male	59	−	427	13	−	82%	+ (GCW)	−	−	2+ (Ms)	−	−	−
5	Male	22	−	269	96	−	84%	−	−	−	−	−	−	−
6	Male	66	−	796	116	+	100%	2+ (GCW)	−	−	−	−	−	−
7	Male	62	+	955	176	+	100%	3+ (GCW)	−	+ (Ms)	3+ (GCW)	+ (GCW)	3+ (GCW)	−
8	Female	30	−	682	60	−	100%	2+ (GCW)	−	+ (Ms)	2+ (Ms)	−	+ (Ms)	−
9	Female	21	+	614	113	−	100%	3+ (GCW)	−	−	−	−	−	−
10	Female	21	+	475	60	−	91%	−	−	−	−	−	+ (Ms)	−

SCr: serum creatinine, ANCA: anti-neutrophil cystoplasmic antibodies, FRA: fibrinogen related antigens, Alb: albumin, GCW: glomerular capillary wall, Ms: mesangium.

This study included two patients with negative staining for linear IgG, possibly because of the severe destruction of glomerular capillary walls. The diagnosis was confirmed by the detection of anti-GBM IgG in serum and linear IgG staining on paraffin tissue by immunofluorescence.

All the 10 patients received renal biopsy with an average of 29.3±12.4 glomeruli seen in each section. All the patients were diagnosed as crescentic glomerulonephritis (over 50% of the glomeruli had larger crescents) with a mean percentage of crescent formation in 85.3±14.6% of the glomeruli. The percentage of crescents in glomeruli was positively correlated with the concentration of serum creatinine at presentation (r = 0.83, *P* = 0.003). Routine direct immunofluorescence showed that linear IgG deposition along GBM was detected in eight (80%) patients with fluorescence intensity +∼3+ on the scale of 0 to 4+. Five (50%) patients had linear or granular C3c deposition along GBM with fluorescence intensity 2+∼3+. Only one patient had mild deposition of C1q along GBM.

### Deposition of Complement Components in Glomeruli

#### C5b-9

**Figure 1:**
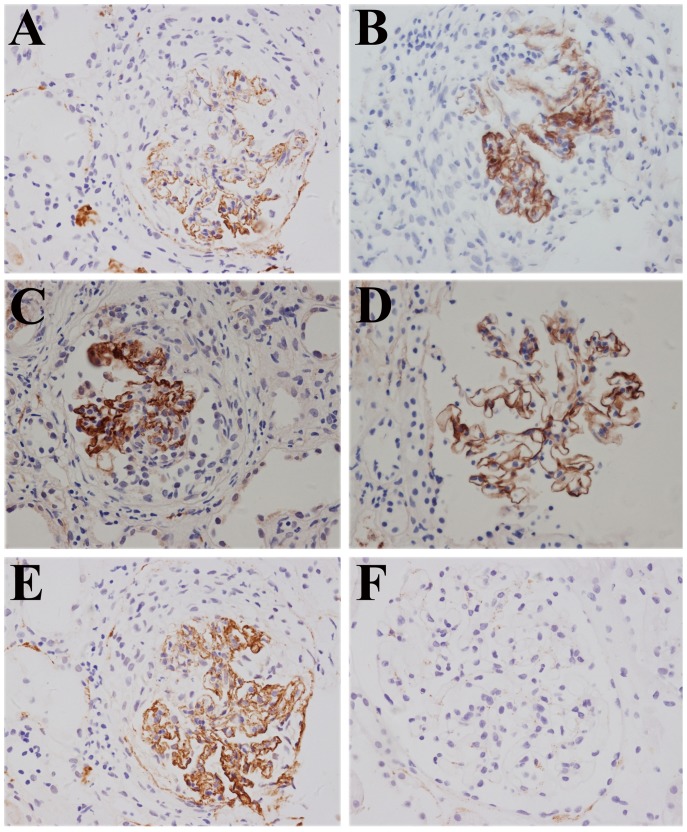
Complement components C5b-9, C3d, C4d, C1q and factor B were detected on paraffin sections of renal tissues from patients with anti-GBM disease by immunohistochemistry (magnification ×400). A: C5b-9 linear deposition along the glomerular capillary wall (Patient #7); B: C3d linear deposition along the capillary wall (Patient #2); C: C4d linear deposition along the capillary wall (Patient #5); D: C1q linear deposition along the capillary wall (Patient #1); E: factor B linear deposition along the capillary wall (Patient #7); F: all the complement components above were negative in the renal tissues from patients with minimal change disease.

C5b-9, also called membrane attack complex, represents the terminal product of complement activation. The number of glomeruli scored per biopsy of paraffin sections was 5.8±2.3 (range 2–9) in immunohistochemistry staining. C5b-9 was detected clearly in a linear pattern along the GCW in 29 (100%) glomeruli of all the 10 patients, and in several glomeruli along the tubular basement membrane (Figure 1A). Occasionally, C5b-9 was found in the mesangial regions or the small artery walls in a mild granular pattern. The intensity of C5b-9 staining was scored as 1 (2/29 glomeruli), 2 (6/29 glomeruli), 3 (15/29 glomeruli) and 4 (6/29 glomeruli) (Table 3). Compared with the glomeruli without crescent formation, the intensity of C5b-9 deposition was much stronger (3.2 vs. 1.6, *P*<0.001) in glomeruli with crescent formation. No deposit of C5b-9 was found in renal biopsy specimens from patients with minimal change disease or normal control renal tissue.

**Table 3:** The deposition of complement components in glomeruli of patients with anti-GBM disease.

Complement	Glomeruli per section (mean±SD)	Glomerular capillary walls, n (%)	Mesangial regions, n (%)	Crescents, n (%)	Bowman's capsules, n (%)	Scores			
						1	2	3	4
C5b-9	5.8±2.3	29/29 (100%)	12/29(41.4%)	0/29(0%)	3/29(10.3%)	2/29	6/29	15/29	6/29
C3d	6.6±3.8	33/33(100%)	7/33(21.2%)	0/29(0%)	0/29(0%)	4/33	4/33	17/33	8/33
C4d	8.8±4.2	50/53(94.3%)	5/53(9.4%)	0/53(0%)	0/53(0%)	6/50	11/50	29/50	4/50
C1q	7.6±3.6	76/76(100%)	13/76(17.1%)	0/76(0%)	55/76(72.4%)	13/76	39/76	20/76	4/76
MBL	3.3±1.0	13/13(100%)	13/13(100%)	9/13(69%)	10/13(76.9%)	0/13	3/13	10/13	0/13
Factor B	4.2±2.3	21/21(100%)	7/21(33.3%)	0/21(0%)	0/21(0%)	2/21	4/21	15/21	0/21
Properdin	3.0±1.0	9/9(100%)	3/9(33.3%)	0/9(0%)	0/9(0%)	0/9	0/9	9/9	0/9

SD: standard deviation, MBL: mannose-binding lectin.

#### C3d

The number of glomeruli scored for C3d per biopsy of paraffin sections was 6.6±3.8 (range 2–10). C3d was detected clearly in a linear or granular pattern along the GCW of 33 (100%) glomeruli of all the 10 patients (Figure 1B). The intensity of C3d was scored as 1 (4/33 glomeruli), 2 (4/33 glomeruli), 3 (17/33 glomeruli) and 4 (8/33 glomeruli) (Table 3). No correlation was found between the intensity of C3d deposition and the clinical characteristics of patients.

#### C4d

The number of glomeruli scored for C4d per biopsy of paraffin sections was 8.8±4.2 (range 4–15). C4d could be detected clearly along the GCW in a linear pattern (Figure 1C). 50/53 (94.3%) glomeruli were positive for C4d deposition, with the intensity scored as 1 (6/50 glomeruli), 2 (11/50 glomeruli), 3 (29/50 glomeruli) and 4 (4/50 glomeruli) (Table 3). We found no correlation between the intensities of C4d deposition and the clinical features of patients.

#### C1q

C1q represents the classical pathway of complement activation. The number of glomeruli scored for C1q per biopsy of paraffin sections was 7.6±3.6 (range 4–13). C1q linear deposition could be found along the GCW and the Bowman's capsules in 76 (100%) glomeruli of all the 10 patients (Figure 1D). The intensity was scored as 1 (13/76 glomeruli), 2 (39/76 glomeruli), 3 (20/76 glomeruli) and 4 (4/76 glomeruli) (Table 3). We found no correlation between the intensities of C1q deposition and the clinical features of patients.

#### MBL

**Figure 2:**
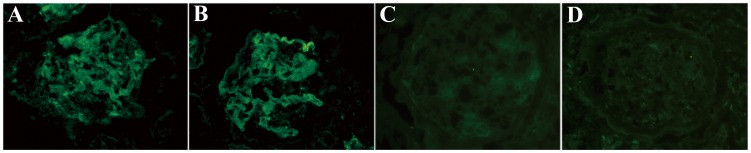
Complement components MBL and properdin were detected on frozen renal tissues from patients with anti-GBM disease by immunofluorescence (magnification ×400). A: MBL diffusive granular deposition on the glomerular capillary wall, mesangial region and crescent in the glomeruli (Patient #4); B: properdin linear deposition along the capillary wall (Patient #10); C: MBL were negative in the renal tissues from patients with minimal change disease; D: Properdin was negative in the renal tissues from patients with minimal change disease.

MBL represents the lectin pathway of complement activation. The number of glomeruli scored for MBL per biopsy of frozen sections was 3.3±1.0 (range 2–4) using immunofluorescence. MBL diffusive deposition could be detected along GCW, in mesangial regions and in crescents in a linear or granular pattern (Figure 2A). 13/13 (100%) glomeruli in frozen sections were positive for MBL deposition, with the intensity scored as 1 (0/13 glomeruli), 2 (3/13 glomeruli), 3 (10/13 glomeruli) and 4 (0/13 glomeruli) (Table 3). We found no correlation between the intensities of MBL deposition and the clinical features of patients.

#### Factor B and Properdin

Factor B and properdin both represent the alternative pathway of complement activation. The number of glomeruli scored for factor B per biopsy of paraffin sections was 4.2±2.3 (range 2–8) using immunohistochemistry. Factor B deposition could be detected along GCW in a linear and granular pattern (Figure 1E). 21/21 (100%) glomeruli were all positive for factor B deposition, with the intensity scored as 1 (2/21 glomeruli), 2 (4/21 glomeruli), 3 (15/21 glomeruli) and 4 (0/21 glomeruli) (Table 3). The intensity of factor B deposition was related with the crescent formation in glomeruli. The intensity of factor B deposition was significantly stronger in the glomeruli with crescent formation (3.3 vs. 1.2, *P*<0.001), compared with the glomeruli without crescent.

The number of glomeruli scored for properdin per biopsy of frozen sections was 3.0±1.0 (range 2–4) using immunofluorescence. Properdin deposition showed a linear deposition on GCW in all patients (Figure 2B). 9/9 (100%) glomeruli were positive for properdin deposition, with the intensity scored as 1 (0/9 glomeruli), 2 (0/9 glomeruli), 3 (9/9 glomeruli) and 4 (0/9 glomeruli) (Table 3). All the glomeruli had crescent formation.

### Co-localization of Complement Components by Immunofluorescence

**Figure 3:**
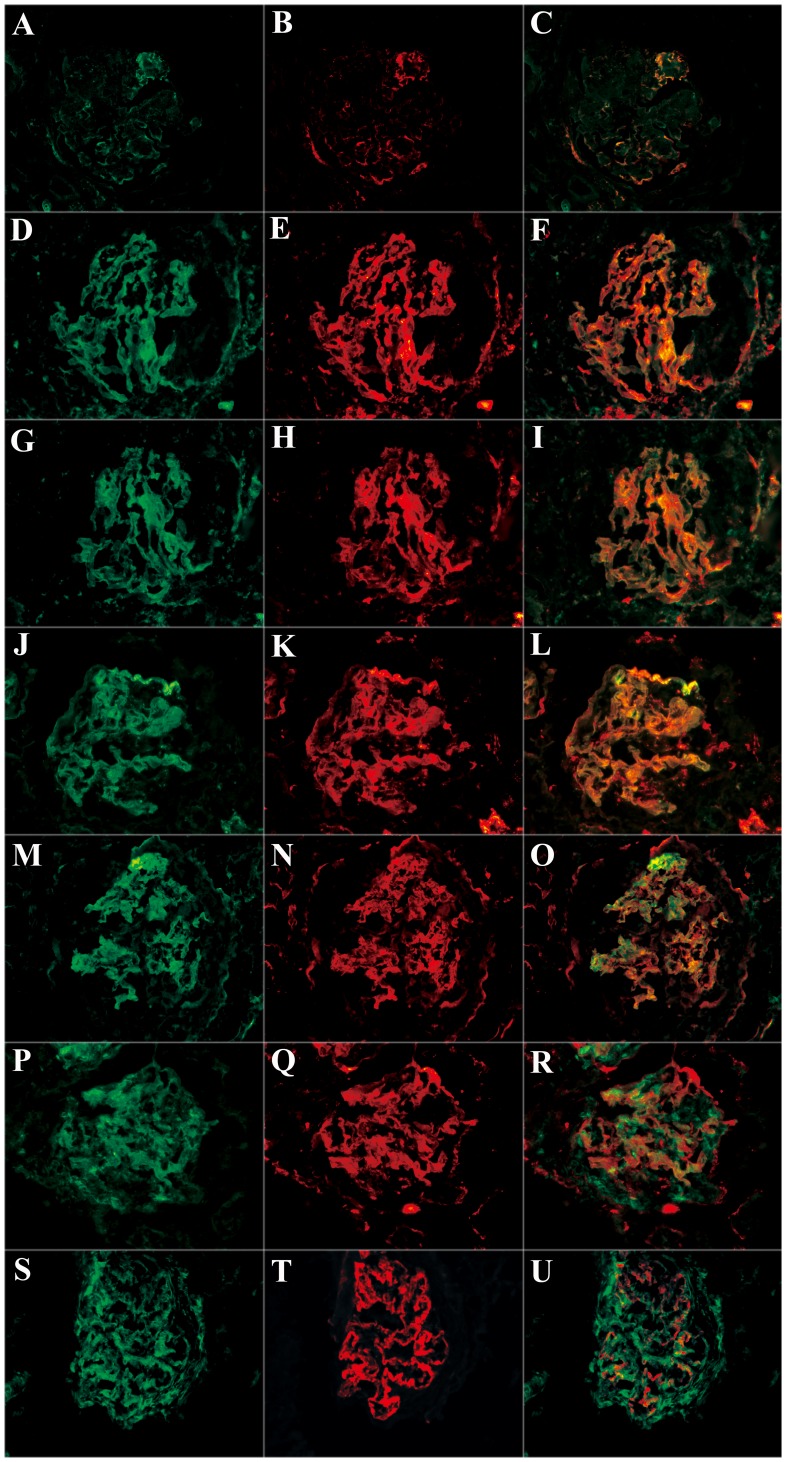
Co-localization of various complement components were detected in frozen sections by immunofluorescence using laser confocal microscopy (magnification ×400). A: IgG linear deposition along the glomerular capillary wall; B: C3d granular deposition along the glomerular capillary wall in the same section; C: IgG and C3d co-localized completely; D: C1q linear deposition along the glomerular capillary wall; E: C5b-9 granular deposition along the glomerular capillary wall in the same section; F: C1q and C5b-9 co-localized completely; G: factor B linear deposition along the glomerular capillary wall; H: C5b-9 granular deposition along the glomerular capillary wall in the same section; I: factor B and C5b-9 co-localized completely; J: properdin linear deposition along the glomerular capillary wall; K: C5b-9 granular deposition along the glomerular capillary wall in the same section; L: properdin and C5b-9 co-localized completely; M: properdin linear deposition along the glomerular capillary wall; N: C3d granular deposition along the glomerular capillary wall in the same section; O: properdin and C3d co-localized completely; P: MBL diffusive deposition; Q: C5b-9 granular deposition along the glomerular capillary wall in the same section; R: MBL and C5b-9 could not co-localize; S: MBL diffusive deposition; T: C4d granular deposition along the glomerular capillary wall in the same section; U: MBL and C4d partially co-localized along the glomerular capillary wall.

By immunofluorescence and laser scanning confocal microscopy, the positive staining of IgG, C5b-9, C4d, C3d, C1q, MBL, factor B and properdin was further confirmed on the glomeruli of patients with anti-GBM disease (Figure 3).

IgG co-localized with C3d exactly along the GCW (Figure 3A–C). C1q, factor B and properdin co-localized with C5b-9 exactly along the GCW too (Figure 3D–L). Furthermore, properdin co-localized with C3d along the GCW (Figure 3M–O). As for MBL, it was found just partially co-localized with C4d (Figure 3S–U), but was not co-localized with C5b-9 at all (Figure 3P–Q).

In this study, we detected all the three pathways of complement activation, which included seven kinds of complement components. Confocal microscopy was also performed for all the three pathways. These examinations consumed lots of sections from the renal biopsy tissues, which made the renal tissues not sufficient for all the detections on all the ten patients. The staining for C1q was performed for all patients, while C4d for six patients, C5b-9, C3d and factor B for five patients, MBL for four patients and properdin for three patients.

## Discussion

In our present study, C3d deposition on glomerular capillary wall was shown in each glomerulus of all patients with anti-GBM disease, even in those C3c negative cases, which indicated that the complement system was activated after the binding of anti-GBM IgG to the target antigens. C3c is in free state and C3d binds covalently via thiolester bond on the membrane of activated site [11]. Thus C3c indicates the ongoing complement activation while C3d can be detected not only at the active lesions but also where after complement activation [12]. In the current study, the general finding of C3d deposition on the capillary wall reflects the activation of complement system during the renal damage process of human anti-GBM disease.

C3 can be activated via the classical, the lectin or the alternative pathway. Classical pathway is initiated with C1q binding to the immune complex. We detected C1q deposition along the glomerular capillary wall in each patient. The same result was found on C4d, the later component of classical activation. Furthermore, C1q deposition co-localized well with C5b-9, the terminal complement complex. These findings indicate the participation of classical pathway in the complement activation of human anti-GBM disease. This was also proven using the C1q knockout mice, in which the kidney injury and C3 deposition caused by the induction of heterologous anti-GBM antibodies were attenuated in comparison with the wild type mice [8]. The absence of C1q in routine direct immunofluorescence on renal biopsy examination is commonly observed, not simply in anti-GBM disease, which may be explained by the following reasons. Unlike C3d and C4d, C1q does not bind covalently to its ligands, which results in its short half-life time *in vivo* and easy to be cleared by macrophages [13]. Also, the lack of anti-C1q antibody in the disease could not display the local stabilizing effect for C1q deposition as it does in lupus nephritis [14].

Factor B and properdin are unique factors needed in the alternative complement pathway. In the present study, we identified the deposition of factor B and properdin along the glomerular capillary wall in each patient. Furthermore, the deposition of factor B and properdin both co-localized well with C5b-9. Properdin could co-localize with C3d as well. These findings indicate the activation of alternative pathway. Bb is a split product after factor B activation, which makes our detection more definite for the indication of alternative complement activation [15]. Properdin is a positive regulation protein, which binds to the C3 convertase, C3bBb, and protects it from the cleavage effects of factor H and factor I [16]. In C4 knockout mice, which lack an intact classical pathway and lectin pathway, the injection of heterologous anti-GBM antibodies could still produce almost the same degree of proteinuria as compared with the wild type mice [8]. These findings provide evidence for the involvement of alternative complement activation in anti-GBM disease.

MBL is the key molecule in the activation of lectin pathway [17], [18], [19]. We did find the deposition of MBL in our patients; however, the staining appeared diffusively, not only on the glomerular capillary wall, but also in the mesangial region, Bowman's capsule and even within the crescents. In addition, MBL could not co-localize with C5b-9 and just partially co-localized with C4d, which indicates that the lectin pathway may be not involved in the complement activation of human anti-GBM disease. The reason for MBL deposition, we suggested, should attribute to its general binding to the carbohydrate ligands, including mannose and N-acetylglucosamine, which were greatly exposed during the glomerular damage of the disease.

In this current study, we found a positive correlation between the intensity of factor B deposition and the crescent formation in glomeruli. The staining intensity of factor B was much stronger in glomeruli with crescents. Our recent study on the circulating complement also found a positive correlation between the plasma level of factor B and the severity of kidney injury. Thus it is implied indirectly that the alternative complement activation might play a pathogenic role in the renal damage of the disease. The pathogenic role of the alternative pathway can only be demonstrated in a GBM model using properdin-deficient and C1q-deficient animals. When properdin-deficient animals show reduced proteinuria and glomerular injury, this may clearly proves that the alternative pathway is pathogenic. However, at this stage this is still for debate.

The alternative pathway might exert the pathogenic role through the following processes. Firstly, the classical pathway is triggered by anti-GBM antibody bindings; then the activation and generation of inflammatory molecules might be further amplified by the alternative pathway [20], [15]. In an arthritis model, Ji et al [21] have shown that the alternative pathway is critical for the recruitment and/or activation of polymorphonuclear leukocytes and development of arthritis, while the classical pathway are entirely dispensable for the effect phase of arthritis. Indeed, the alternative pathway may participate in 80% of total complement activation and be both required and sufficient for tissue injury [22], [23], [24]. Secondly, properdin itself can act as the pathogen recognition protein, binds to the apoptotic cells and target surfaces, and then initiates and amplifies the alternative complement activation [25], [26], [27], [28]. All these characteristics may contribute to the pathogenic role of alternative pathway in the kidney injury of human anti-GBM disease.

In conclusion, the present study demonstrates that the complement system is overall activated, via the classical pathway and the alternative pathway, in the kidney of human anti-GBM disease. The alternative complement activation might play a pathogenic role in the process of renal damage.
